# Intravascular Wrap for Treatment of Basilar Artery Perforator Aneurysm

**DOI:** 10.7759/cureus.18021

**Published:** 2021-09-16

**Authors:** Anna Luisa Kuhn, Ajit S Puri, Francesco Massari, Jasmeet Singh

**Affiliations:** 1 Division of Neurointerventional Radiology, Department of Radiology, University of Massachusetts Medical Center, Worcester, USA

**Keywords:** snuffbox approach, subarachnoid hemorrhage, flow diverter, distal radial artery, cangrelor, basilar artery, aneurysm

## Abstract

Basilar artery perforator aneurysms are very rare and usually present with subarachnoid hemorrhage. High-quality imaging systems with digital subtraction angiography and three-dimensional rotational angiography are crucial for the detection of these posterior circulation vascular lesions, which may still be missed on the initial angiogram. We present the first use of a Flow Re-Direction Endoluminal Device (Microvention, Aliso Viejo, California) for treatment of a ruptured basilar artery perforator aneurysm via snuffbox vascular access and use of cangrelor for antiplatelet management.

## Introduction

Perforator aneurysms of the intracranial vasculature are rare but basilar artery perforator aneurysms (BAPAs) are even rarer (less than 1%) [1]. Not much is known about their natural history and there is no consensus on the management of these aneurysms. The literature is somewhat divided between surgical obliteration, endovascular treatment, or conservative management [2]. Although spontaneous thrombosis of BAPAs was reported in the literature, there is a rebleeding risk of 11% to 15% in untreated patients [3]. Among endovascular treatment options, coiling is preferred. However, microcatheter navigation into the perforator may be difficult, if not impossible, given the small vessel or aneurysm size and/or unfavorable angle between the basilar artery and perforator origin. Flow diversion may be a valid alternative but the risk of thromboembolic complications and the need for dual antiplatelet therapy must be considered [4,5]. Here, we describe the first use of a Flow Re-Direction Endoluminal Device (FRED; Microvention, Aliso Viejo, California) for the treatment of a ruptured BAPA via snuffbox (distal radial artery) vascular access and use of cangrelor for antiplatelet management.

## Case presentation

**Figure 1:**
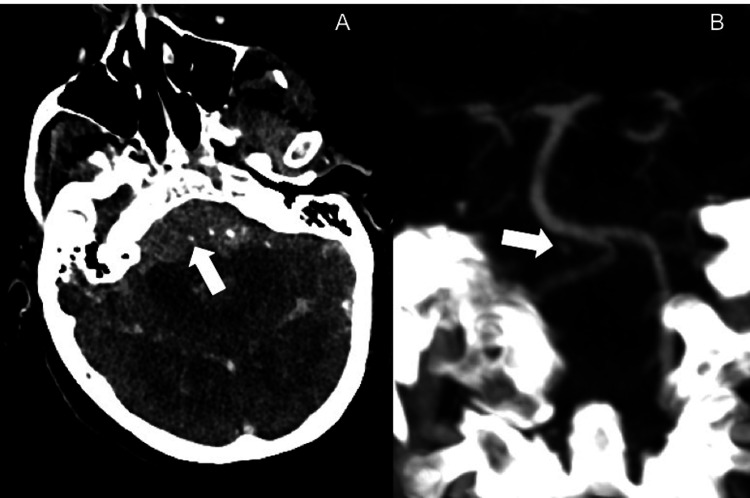
Ruptured basilar perforator aneurysm on CT. Axial (A) and coronal (B) CT angiogram images showing focal contrast pooling adjacent to the proximal basilar artery.

A patient presented to our emergency room with acute severe headache and loss of consciousness (Glasgow Coma Scale of 3). The patient was found to have extensive subarachnoid hemorrhage, predominantly perimesencephalic and at the craniocervical junction on non-contrast head CT with intraventricular extension and mild hydrocephalus (modified Fisher scale of 4) (Figure 1A). CT angiogram revealed a focus of contrast pooling adjacent to the proximal basilar artery, suspicious for a ruptured BAPA (Figure 1B).

**Figure 2:**
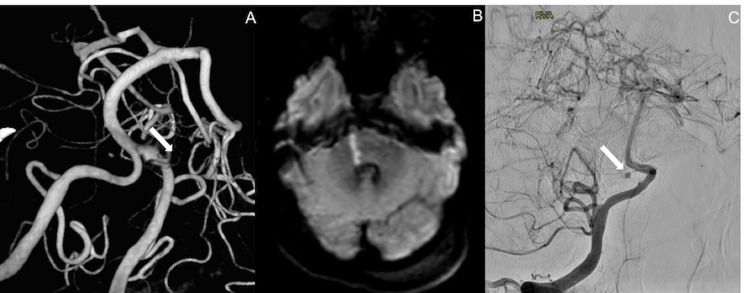
Ruptured basilar perforator aneurysm and infarct. Three-dimensional rotation angiography (A) demonstrates the focal contrast pooling, which was not seen on the initial angiogram. MRI shows a small basilar perforator stroke (B). A follow-up angiogram (C) confirms the presence of a basilar artery perforator aneurysm.

An external ventricular drain was placed (maintained at 15 mmHg), and the patient underwent a cerebral angiogram. The initial cerebral angiogram did not show the aneurysm. It was only visible on the three-dimensional rotational angiogram but no connection to the basilar artery was identified (Figure 2A). The patient’s exam initially improved after external ventricular drain placement with the ability to follow intermittent commands. Unfortunately, the patient’s exam then declined the following evening, and an urgent MRI showed a focus of diffusion restriction in the right pons. Given that the MRI only showed a small perforator stroke, the multidisciplinary team sought to give the patient a chance to recover even with a poor clinical exam. We offered endovascular repair of the BAPA.

The patient was brought back for a repeat angiogram via six French snuffbox vascular access, which revealed that the aneurysm was arising from a proximal right basilar artery perforator (Figures 2B, 2C).

**Figure 3:**
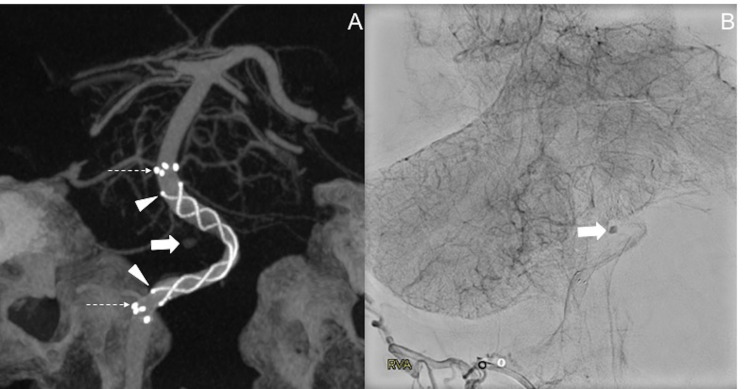
Endovascular treatment of the ruptured basilar perforator aneurysm. Three-dimensional reconstruction of a Vaso-CT (A) performed after placement of the Flow Re-Direction Endoluminal Device (FRED) shows the distal device markers indicating the end of the flare tips of the device (dotted arrow), the central flow diversion portion of the device (arrowheads), and the location of the perforator aneurysm (thick arrow). Delayed images of the angiogram post device deployment (B) show contrast stagnation within the basilar artery perforator aneurysm.

This vessel, however, was too small for direct catheterization. After weighing all options, the decision was made to place a flow diverter. Among the devices available, a 4 x 23 mm FRED was chosen and deployed via a Headway 27 microcatheter (Microvention, Aliso Viejo, California) (Figure 3A). A Benchmark guide catheter (Penumbra, Alameda, California) and five French Sofia (Microvention, Aliso Viejo, California) distal access catheters were placed in the distal V2 segment and within the V4 segment of the right vertebral artery, respectively, which supported the delivery of the flow diverter. Delayed angiogram after device deployment showed contrast stagnation within the aneurysm (Figure 3B).

The patient was given an intraprocedural 30 mcg/kg intravenous bolus of cangrelor at the time of flow diverter placement with a 2 mcg/kg/min intravenous drip post-procedure.

We did not observe any intraprocedural thromboembolic complications. Follow-up imaging a few hours postoperatively and on post-operative day five showed no new bleed. Follow-up CT angiogram and MRI on post-operative days five and seven, respectively, showed a patent device without new infarcts. Unfortunately, the patient did not improve neurologically and the family withdrew care after 12 days.

## Discussion

BAPAs are challenging to diagnose and treat. These aneurysms are often small, possibly partially thrombosed, and slow filling, which makes them difficult to visualize on angiography. Thus, repeat angiography, such as in our case, is often necessary to make the finding [4]. The use of flow diversion has been proposed as a possible treatment option for BAPAs but basilar perforator strokes are a feared complication [5]. Also, the need for intravenous antiplatelet medication after emergent flow diversion may be a reason why some interventionalists hesitate to place a flow diverter in the acute setting, particularly in a ruptured aneurysm case. In certain cases, however, flow diverters represent a valuable treatment option, and we feel our case supports their use. It is important to note, however, that for BAPAs, the use of flow diverters is off-label. Nevertheless, another flow diverter off-label uses such as for treatment of ruptured blood blister or dissecting aneurysms have shown to be a valid treatment alternative [6,7] and, in some cases where conventional endovascular or neurosurgical treatments can be challenging, placement of a flow diverter may actually be the safer choice.

Our case addresses three major concerns of a neurointerventionalist when deciding on flow diversion treatment of a ruptured aneurysm: (1) choice of a safest and most effective device; (2) best antiplatelet management; and (3) lowest risk of access site complications.

The FRED device is a dual-layer, self-expanding braided nitinol mesh flow diverter. The flared ends of the outer layer extend on each side by about 3 mm with no flow-diverting property, making it a great option in perforator-rich areas. The dual-layer, flow diverting portion of the device can be positioned accurately over the aneurysm, whereas the flared ends are able to anchor the device within the parent vessel without compromising flow into branch vessels/perforators. This is particularly important along the basilar artery as the device decreases the coverage of uninvolved perforators. Other flow diverters do not have this special design and would cover more perforators along the entire length of the device. In our case, we were able to anchor the FRED just at the level of the anterior inferior cerebellar artery origins and spanning proximally to the origin of the right posterior inferior cerebellar artery. Because of the flared, single-layer ends of the device, there is no flow compromise into these branches. The dual-layer, flow diverting portion of the device was positioned along the proximal basilar and distal V4 segment of the right vertebral artery, excluding the blood flow to the BAPA, which shows contrast stasis on the delayed view of the angiogram after device placement. On follow-up MRI, there were no new procedure-related infarcts. Despite our successful use of the FRED, it is important to note that aneurysm occlusion is usually delayed, which may leave the patient at risk for re-rupture, especially in the acute phase [4]. Nevertheless, BAPAs are considered slow filling lesions that should have a lower risk of re-rupture when compared to other aneurysms [8,9].

Antiplatelet management was started at the time of device placement so that the patient would only be exposed to the re-bleeding risk at the time of definitive treatment. Cangrelor is the only intravenous P2Y12 platelet inhibitor approved by the Food and Drug Administration. It has a shorter onset of action (two minutes), rapid achievement of steady-state concentrations, and a shorter offset of action (one hour) compared to other oral P2Y12 inhibitors, which allow for rapid restoration of platelet function. Neuroendovascular data on the safety of cangrelor use in the acute setting have been described in the past couple of years [10,11]. More information on patients with intracranial bleeds and cangrelor use is needed; however, cangrelor possesses the best safety profile of the drugs currently available, making it the best choice of antiplatelet therapy needed in the setting of a ruptured aneurysm. There was no new bleed in our patient on follow-up imaging post-procedure.

Given the need for dual antiplatelet therapy after flow diversion (one agent lifelong and the other for at least six months), our patient would already be at risk for (re-)bleeding and we intended to minimize any additional risk for another complication related to vascular access. Therefore, the procedure was performed via a distal radial artery (snuffbox) access. When comparing transfemoral versus wrist access, it is known that transfemoral interventions carry a higher risk of access site and bleeding complications, greater in-hospital mortality rates, and higher costs [12]. Choosing snuffbox over radial access was due to the advantage of decreased risk for hematoma formation, lower risk of compartment syndrome, and ability to save the radial artery for any future interventions [13]. Our patient did not experience any vascular access site complications.

## Conclusions

Treatment of (ruptured) BAPAs is a challenging procedure for neurointerventionalists. Flow diversion may be an appropriate treatment option in selected patients. Further considerations regarding the vascular access site and antiplatelet management are important to formulate the best possible treatment plan. A combination of snuffbox vascular access, FRED, and cangrelor currently represents one of the best treatment strategies for (ruptured) BAPAs.
